# Early detection and intervention using neutrophil gelatinase-associated lipocalin (NGAL) may improve renal outcome of acute contrast media induced nephropathy: A randomized controlled trial in patients undergoing intra-arterial angiography (ANTI-CIN Study)

**DOI:** 10.1186/1471-2369-12-39

**Published:** 2011-08-17

**Authors:** Gernot Schilcher, Werner Ribitsch, Ronald Otto, Rupert H Portugaller, Franz Quehenberger, Martini Truschnig-Wilders, Robert Zweiker, Philipp Stiegler, Marianne Brodmann, Klemens Weinhandl, Joerg H Horina

**Affiliations:** 1 Division of Nephrology and Hemodialysis, Department of Internal Medicine, Medical University of Graz, Austria; 2 Department of Radiology, Medical University of Graz, Austria; 3 Institute for Medical Informatics, Statistics and Documentation, Medical University of Graz, Austria; 4 Clinical Institute for Laboratory Medicine, Medical University of Graz, Austria; 5 Division of Cardiology, Department of Internal Medicine, Medical University of Graz, Austria; 6 Division of Transplantation Surgery, Department of Surgery, Medical University of Graz, Austria; 7 Division of Angiology, Department of Internal Medicine, Medical University of Graz, Austria

## Abstract

**Background:**

Patients with pre-existing impaired renal function are prone to develop acute contrast media induced nephropathy (CIN). Neutrophil gelatinase-associated lipocalin (NGAL), a new biomarker predictive for acute kidney injury (AKI), has been shown to be useful for earlier diagnosis of CIN; however, urinary NGAL values may be markedly increased in chronic renal failure at baseline. Results from those studies suggested that urinary NGAL values may not be helpful for the clinician. An intravenous volume load is a widely accepted prophylactic measure and possibly a reasonable intervention to prevent deterioration of renal function. The aim of our study is to evaluate NGAL as an early predictor of CIN and to investigate the clinical benefit of early post-procedural i.v. hydration.

**Methods/Design:**

The study will follow a prospective, open-label, randomized controlled design. Patients requiring intra-arterial contrast media (CM) application will be included and receive standardized, weight-based, intravenous hydration before investigation. Subjects with markedly increased urinary NGAL values after CM application will be randomized into one of two study groups. Group A will receive 3-4 ml/kg BW/h 0.9% saline intravenously for 6 hours. Group B will undergo only standard treatment consisting of unrestricted oral fluid intake. The primary outcome measure will be CIN defined by an increase greater than 25% of baseline serum creatinine. Secondary outcomes will include urinary NGAL values, cystatin C values, contrast media associated changes in cardiac parameters such as NT-pro-BNP/troponin T, changes in urinary cytology, need for renal replacement treatment, length of stay in hospital and death.

We assume that 20% of the included patients will show a definite rise in urinary NGAL. Prospective statistical power calculations indicate that the study will have 80% statistical power to detect a clinically significant decrease of CIN of 40% in the treatment arm if 1200 patients are recruited into the study.

**Discussion:**

A volume expansion strategy showing a benefit from earlier intervention for patients with markedly elevated urinary NGAL values, indicating a CIN, might arise from data from this study.

**Trial registration:**

ClinicalTrials.gov NCT01292317

## Background

In recent decades, the growing demand for sophisticated diagnostics in all fields of modern medicine has increased the need for contrast-enhanced imaging [1]. The amount of iodinated contrast media (CM) used worldwide cannot be even roughly estimated. While contrast-enhanced procedures are fairly safe in the healthy population, patients with pre-existing impaired renal and/or cardiac function are prone to develop acute kidney injury (AKI) due to acute contrast induced nephropathy (CIN) and so have a greater risk of death [2,3]. This has been shown in patients undergoing coronary angiography and intervention who sometimes end up with end-stage renal disease [4,5]. The effect of an immediate post-procedural hemodialysis treatment to eliminate CM load is also discussed controversially [6,7].

Until recently and for this reason, physicians have increasingly used gadolinium enhanced magnetic resonance imaging to avoid renal complications, especially in such high-risk patients. For interventional settings, however, gadolinium enhanced magnetic resonance imaging has never been an appropriate alternative.

Also, with the recognition of nephrogenic systemic sclerosis as a severe and also life-threatening gadolinium side effect without treatment options, physicians now have to weigh the risk of either investigation for the individual patient [8,9]. A return to iodinated CM enhanced investigations is now seen in many clinical settings.

A variety of investigators have tried to establish risk scores for patients before investigation [10,11]. To date, a reliable laboratory value or test that recognizes acute renal damage before serum creatinine increases is still being sought and might be a most helpful tool to initiate proper treatment on time. Neutrophil gelatinase-associated lipocalin (NGAL), a new biomarker predictive for acute renal injury, has been shown to be capable of earlier diagnosis in patients undergoing cardiac surgery and CIN [12-16]. NGAL so might be the desired diagnostic link between acute kidney damage and the later occurrence of elevated creatinine values. Randomized controlled trials using this "timesaver" for earlier intervention have not yet been performed, but would be highly desirable [17]. Such a study might change physicians' therapeutic strategies and improve clinical outcomes.

One major drawback of this new biomarker may be the fact that only little is known about its value and accuracy in patients with underlying chronic kidney disease (CKD), and a predictive pattern or cut-off level in patients developing "acute on chronic renal failure" has not yet been defined. Some investigators found markedly increased NGAL levels in individual patients, far above the proposed cut-offs for acute renal failure. They also discovered a significant inverse relation between NGAL and renal function defined by glomerular filtration rate (GFR) [18,19]. Mori et al. have speculated that constantly increased, yet stable NGAL values in CKD are the consequences of sustained production by "inflamed", but vital tubular cells [20]. Even more complicating is the fact that age and several co-morbidities, such as inflammation and chronic heart disease, common in patients with chronic renal failure, may also influence NGAL levels [21,22]. A meta-analysis by Haase et al. selected 19 relevant published articles and defined 150 ng/ml as a possible cut-off value with a proper sensitivity and specificity [23]. This analysis also confirmed a comparable accuracy of plasma and urinary NGAL, whereas urinary NGAL testing may be even better in patients with CKD [24].

So far, an intravenous volume load is the only reasonably proven and widely accepted prophylaxis for CIN after CM application [25-28]. In the majority of the relevant randomized controlled trials, isotonic saline infusion (0.9%) was used for volume expansion, while some authors used half-isotonic saline (0.45%) or sodium bicarbonate at higher volumes. The benefit of this type of intervention is not a pharmacologic effect of a specific type of infusion, but rather a physical effect of volume expansion. The optimal intravenous fluid regimen regarding the type, amount, route and duration of volume application remains controversial.

Hydration regimens have differed widely among relevant randomized trials and so are not comparable [29]. Further, most studies also lacked statistical power, used different types of CM and definitions of CIN or allowed for additional prophylactic measures, such as N-acetylcysteine or sodium bicarbonate in a varying percentage of their patients [30-38].

Worldwide, there are no generally accepted guidelines for a controlled and standardized prophylactic volume application among radiologists, cardiologists, nephrologists and other involved physicians. In other words, the beneficial effect of intravenous hydration is not used to greatest advantage.

In our study, all patients requiring intra-arterial CM application will receive a weight-based, standardized intravenous hydration before investigation. This measure of controlled pre-hydration alone might be of great benefit for our patients and differs from routine clinical practice, where patients often only receive oral fluid at best. To date, intravenous hydration is not even an established clinical standard for the so-called "high-risk patient" with e.g. diabetes and/or renal failure.

A randomized trial evaluating the clinical benefit of additional post-procedural i.v. hydration might provide a further benefit and could be highly valuable for routine practice.

Controlled post-procedural i.v. hydration has not been used consistently for several reasons [29]:

(a) worldwide, a large percentage of CM studies are performed on an outpatient basis;

(b) there are no clear data proving the benefit of additional post-procedural i.v. hydration, versus pre-procedural hydration only;

(c) intravenous hydration, both pre- and post-procedural, is often not performed for organizational reasons.

### Rationale and hypothesis

Earlier detection of CIN by the novel urinary NGAL test method enables amelioration of renal injury by the introduction of rapid and intensified post-procedural (angiography) volume expansion.

### Aims of our study

1). To evaluate the true risk of CIN in high-risk patients following intra-arterial angiography using a standardized pre-procedural protocol for intravenous volume load.

2). To evaluate the benefit of an earlier diagnosis of renal damage after investigation using urinary NGAL.

3). To investigate the effect of a well-timed and intensified post-procedural volume expansion on renal function, morbidity and mortality in patients predicted to develop CIN according to NGAL testing.

4). To study NGAL in patients with mild, moderate and severe CKD (stage 2-4), a subgroup of individuals who frequently develop CIN. At present it is still unclear whether the new biomarker NGAL is comparably effective in this particular group.

5). To define and establish a reliable cut-off value or pattern of increase for NGAL in patients with chronic renal failure and to estimate its sensitivity and specificity.

6). To assess the value of a 25% increase in serum creatinine as a definition for clinically relevant CIN.

7). To compare the value of NGAL and cystatin C in predicting CIN.

8). To compare the value of the MDRD, the CKD-EPI- and the Cockcroft-Gault formula in this particular patient group.

At this stage, little is known about the beneficial effect of earlier diagnosis and subsequent intervention by volume expansion on AKI due to a rise in urinary NGAL. As the time course of kidney damage is usually not apparent, contrast media induced nephropathy (CIN) is a promising condition for this question. Results from previous studies are unable to guide the clinician when baseline urinary NGAL levels exceed the normal range.

To our knowledge, this is the first clinical study to address this problem. We are trying to assess the effect of an early intervention by volume expansion in patients with increasing urinary NGAL levels 4-6 hours following CM application. If a significant reduction of CIN as defined by creatinine levels is found in the treatment arm within 2 days, a clear benefit of measuring urinary NGAL might be postulated. We are currently recruiting patients for this non-commercial investigator initiated trial to elucidate CIN.

## Methods and design

### Study Design and Setting

This study is a single center, open label, randomized controlled trial involving subjects undergoing an intra-arterial angiographic procedure with iodinated CM. The study is being conducted at the University Hospital in Graz, Austria.

### Ethical considerations

The Ethics Committee of the Medical University of Graz, Austria, approved this trial (Registration Number: 21-278 ex 09/10). The Ethics Committees will be provided with annual reports of trial progress and will promptly receive all adverse event reports. Participants may declare their withdrawal at any time during the study.

### Participants

Patients scheduled for diagnostic intra-arterial catheter angiography or endovascular intervention will be invited to participate in the study. Only one type of iso-osmolar, non-ionic, monomeric CM (Iomeron^®^) will be used. The most common underlying diseases in these individuals are coronary heart disease, peripheral arterial occlusive disease and renal artery stenosis.

### Identification of eligible patients

**Figure 1:**
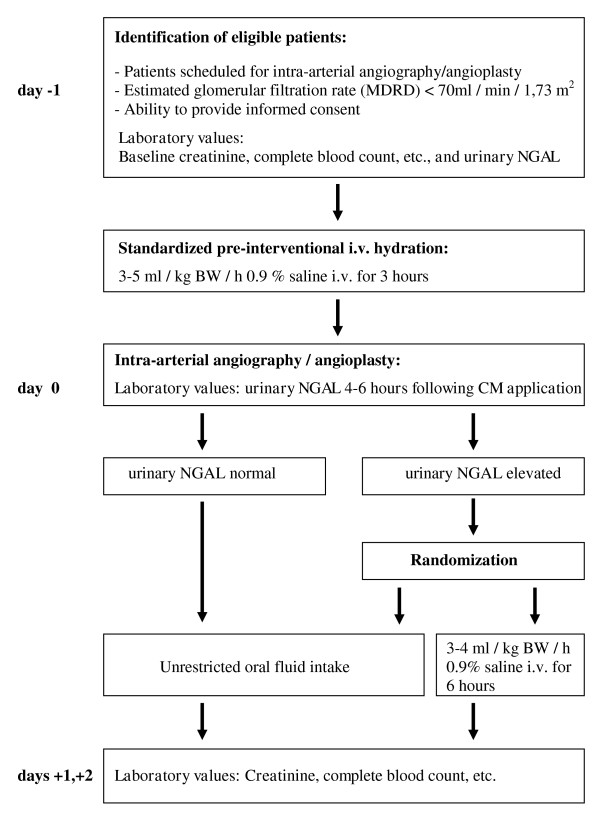
**Scheme for ANTI-CIN Study**.

The principle investigator and the clinical trial coordinators will screen the medical records of patients for pre-existing renal failure one day prior to an angiographic intervention at the Divisions of Nephrology, Cardiology, Angiology and Radiology at the University Hospital in Graz, Austria. Eligible patients will have a copy of the patient information sheet placed in the medical record. The investigator will explain the study during the clinical consultation. Only subjects giving written informed consent will be included. The study period will last 4 days, starting one day prior to investigation and ending two days after the intra-arterial angiography (Figure 1). A one year follow up consultation is targeted. All patients with CIN will be followed separately.

### Inclusion criteria

• Patients requiring intra-arterial angiography/angioplasty

• Patients older than 18 years

• Patients with clinically stable CKD of stage 2 or more (calculated GFR < 70 ml/min/1,73 m2)(65)

• Gender: both

• Written informed consent

### Exclusion criteria

• Pre-existing clinical and/or laboratory evidence of acute renal failure at the time of enrolment

• Evidence of rhabdomyolysis

• Patients on renal replacement therapy (34)

• Patients with life-threatening underlying disease

• Contraindication for volume therapy

• Pregnancy

• CM application within 7 days prior to intervention

### Standardized procedure

Hospitalized patients scheduled for elective intra-arterial angiography will be prepared for CM application using a weight-based hydration protocol.

Monomeric Iomeron^® ^(iomeprol, Bracco Austria, Vienna) will be used exclusively as contrast media. Blood samples for standard evaluation, including serum creatinine and cystatin C, will be drawn at baseline and after 24 and 48 hours from all patients. A BW/h saline infusion (3-5 ml/kg) will be given for 3 hours before CM application. Urine samples for NGAL testing will be collected shortly before (baseline) and after 4 to 6 hours following CM application. NGAL will be measured with the commercially available ARCHITECT^® ^NGAL Test, Abbott Laboratories, Abbott Park, Illinois, U.S.A. [15,39].

Patients who develop CIN will be treated and followed at the Division of Nephrology and Hemodialysis according to established standards.

### Randomization

Patients will be allocated randomly using a Web-based computer program http://www.randomizer.at. Balance of treatment counts is achieved by the biased-coin method with probability 0.7 [40]. Randomization will occur shortly after receipt of the second urinary NGAL value. NGAL determinations will be made by the medical technical staff of the Clinical Institute for Laboratory Medicine, Medical University of Graz, Austria. The criteria for randomization (after 2^nd ^NGAL testing, 4 -6 hours following CM application) are:

• Patients with NGAL levels > 150 ng/ml, if baseline was below 75 ng/ml [23].

• Patients with doubling of NGAL values, if baseline values were > 75 ng/ml [18].

### Procedure following randomization

Study patients with a defined increase of urinary NGAL values at 4 to 6 hours after CM application will be randomized into one of two groups:

Patients of **Group A **will receive 3-4 ml/kg BW/h 0.9% saline intravenously for 6 hours post procedure in addition to oral fluid.

Patients of **Group B **will receive unrestricted oral fluid. An intake of at least 500 ml tea or water is advised and provided on the ward.

### Blinding

Blinding of investigators and patients is not feasible as it is immediately recognizable and impossible to conceal that only one group (Group A) receives saline intravenously.

### Outcome measures

#### Primary outcome measure

CIN defined by an increase greater than 25% of baseline serum creatinine. Additionally, patients will be classified according to the Cockcroft and Gault, as well as the MDRD and the CKD-EPI calculation formula.

#### Secondary outcome measures

Urinary NGAL values, cystatin C values, contrast media associated changes of cardiac parameters such as NT-pro-BNP/Troponin T, changes in urinary cytology, need for renal replacement treatment, length of hospital stay and death.

#### Monitoring for adverse events

The study investigators and the Coordination Center for Clinical Studies, Medical University of Graz, authored a standardized operating procedure for reporting adverse events. Although this trial has not been declared as a pharmacological interventional study, because the volume expansion is considered a physical rather than a pharmacological effect, the investigators voluntarily chose to report adverse events according to the guidelines for a pharmacological interventional study. All relevant adverse events will be summarized and reported to the Ethics Committee of the Medical University of Graz.

### Data collection

Historical data will be obtained from the patient or collected from medical records. These include age, gender, weight, height, relevant co-morbidities, existence of peripheral edema, current medication, contrast media dose and location of angiography/angioplasty/stent implantation. Furthermore, there will be repeated lab studies including complete blood count, electrolytes, serum creatinine, coagulation profile, liver function test, C-reactive protein, bicarbonate and spot urine analysis and urinary NGAL. Length of hospitalization will documented. All data are stored in an electronic case report form.

### Sample size calculations

Sample Size: Alpha = 0.05, power = 0.8, two-sided test. Assuming a clinically relevant difference in CIN incidence of 20% and 60% CIN in the control group (worst case), 108 patients per group are required for the chi square test. Only patients who meet the NGAL criteria for randomization will be included. We assume that 20% of the included patients, which would amount to about 1200 patients, will be randomized to treatment groups. If fewer than 48 patients are randomized after the inclusion of 240 patients, the NGAL criteria for randomization will be adapted in order to meet the objective of including not more than 1200 patients. Recruitment will continue until 240 patients have been randomized.

Analysis: Chi square tests will be used for CIN reduction. Wilcoxon's rank sum test will be applied to continuous measurements.

### Statistical considerations

The diagnostic value of a two-point-measurement of NGAL, cystatin C and creatinine before and after CM application, as compared to a single-point-measurement only after CM application with respect to CIN will be assessed through receiver operating characteristic (ROC) within the patients who received standard therapy. The analysis has to account for the bias that is caused by the exclusion of patients who receive treatment A from ROC analysis on the condition of an elevated NGAL. The available sample size for ROC analysis is determined by the number of patients, the incidence of CIN and the number of patients who receive treatment A. We assume that 20% of the included patients will develop CIN.

### Data analysis

Treatments will be compared according to relative change of creatinine from baseline by the Wilcoxon test and Pearson's chi square test (two-sided, alpha = 0.05).

As mentioned above, those patients without a relevant increase of NGAL will also be followed. This will allow us to retrospectively calculate sensitivity and specificity of NGAL testing for CIN.

## Discussion

To the best of our knowledge there is no other interventional study published or ongoing using "timesaving" effect due to NGAL evaluation to prevent CIN http://www.clinicaltrials.gov.

Though a controlled study evaluating the benefit of early diagnosis and intervention would be highly desirable, the planning of such a study has some major difficulties and drawbacks:

1. An established cut-off for NGAL has not been defined for patients with CKD. Many investigators have found a wide range from approximately 5 to 800 ng/ml in such patients. We therefore believe that at present, one single-point measurement may be insufficient to differentiate between acute and chronic disease and so will perform 2-point measurements. It is hoped that inter-individual changes before and after investigation will be of great help in this differentiation.

2. As an increase of NGAL after cardiopulmonary bypass in children (without pre-existing renal disease or nephrosclerosis) has been found without development of AKI, a mild increase of NGAL values will not lead to randomization [15].

3. Since increased baseline values above the established cut-off for AKI should not lead to exclusion from the study of patients with CKD, we have developed subtle criteria for randomization. It is our hypothesis that at least doubling of NGAL should be demanded in patients with CKD, whereas in patients with very low values at baseline even a higher-fold increase might be without clinical relevance, as long as values stay below 150 ng/ml.

4. To define study size and patient numbers it would be necessary to know the percentage of patients who will show NGAL increase. In such a high-risk patient group, we would estimate that more than 15 to 20 percent of patients might develop CIN, based on the contrast media literature. A fine-tuning of cut-off levels after intermediate analysis would be statistically possible without patient exclusions.

5. The effect of intensified hydration is not clearly established, although data support its benefit.

6. Urinary NGAL seems to be even better than plasma NGAL. The Architect kit showed reliable results [15].

In summary, a clearly defined and simplified prophylactic volume expansion strategy showing a benefit of earlier intervention in patients with markedly elevated urinary NGAL values, indicating CIN, might arise from the data this study produces.

## List of abbreviations

CM: contrast media; AKI: acute kidney injury; CIN: contrast media induced nephropathy; NGAL: neutrophil gelatinase-associated Lipocalin; CKD: chronic kidney disease

## Competing interests

The authors declare that they have no competing interests.

## Authors' contributions

JHH is the principle investigator of this non-commercial investigator initiated study. JHH, GS, WR, RO and KW are responsible for the design of this clinical trial, the construction of the protocol and writing of the manuscript. FQ provided the sample size calculation and the randomization process and critically reviewed the study design and the protocol in terms of statistical aspects. He will also perform the data analysis. HP, MTW, RZ, PS and MB took part in initiating the study, helped to draft the manuscript as experts in their fields and are currently part of the trial managing team. All authors read and approved the final manuscript.

## Pre-publication history

The pre-publication history for this paper can be accessed here:

http://www.biomedcentral.com/1471-2369/12/39/prepub
